# 'Mitochondrial energy imbalance and lipid peroxidation cause cell death in Friedreich's ataxia'

**DOI:** 10.1038/cddis.2016.111

**Published:** 2016-05-26

**Authors:** R Abeti, M H Parkinson, I P Hargreaves, P R Angelova, C Sandi, M A Pook, P Giunti, A Y Abramov

**Affiliations:** 1 Ataxia Centre, Department of Molecular Neuroscience, UCL, Institute of Neurology, Queen Square, London, UK; 2 National Hospital, Neurometabolic Unit, London UK; 3 Department of Molecular Neuroscience, UCL, Institute of Neurology, Queen Square, London, UK; 4 Ataxia Research Group, Division of Biosciences, Department of Life Sciences, College of Health & Life Sciences, and Synthetic Biology Theme, Institute of Environment, Health & Societies, Brunel University London, Uxbridge, UK

## Abstract

Friedreich's ataxia (FRDA) is an inherited neurodegenerative disease. The mutation consists of a GAA repeat expansion within the *FXN* gene, which downregulates frataxin, leading to abnormal mitochondrial iron accumulation, which may in turn cause changes in mitochondrial function. Although, many studies of FRDA patients and mouse models have been conducted in the past two decades, the role of frataxin in mitochondrial pathophysiology remains elusive. Are the mitochondrial abnormalities only a side effect of the increased accumulation of reactive iron, generating oxidative stress? Or does the progressive lack of iron-sulphur clusters (ISCs), induced by *reduced frataxin*, cause an inhibition of the electron transport chain complexes (CI, II and III) leading to reactive oxygen species escaping from oxidative phosphorylation reactions? To answer these crucial questions, we have characterised the mitochondrial pathophysiology of a group of disease-relevant and readily accessible neurons, cerebellar granule cells, from a validated FRDA mouse model. By using live cell imaging and biochemical techniques we were able to demonstrate that mitochondria are deregulated in neurons from the YG8R FRDA mouse model, causing a decrease in mitochondrial membrane potential (▵Ψ_m_) due to an inhibition of Complex I, which is partially compensated by an overactivation of Complex II. This complex activity imbalance leads to ROS generation in both mitochondrial matrix and cytosol, which results in glutathione depletion and increased lipid peroxidation. Preventing this increase in lipid peroxidation, in neurons, protects against in cell death. This work describes the pathophysiological properties of the mitochondria in neurons from a FRDA mouse model and shows that lipid peroxidation could be an important target for novel therapeutic strategies in FRDA, which still lacks a cure.

Friedreich's ataxia (FRDA) is an autosomal recessively inherited neurodegenerative disorder, characterised by progressive spinocerebellar ataxia, together with cardiomyopathy, scoliosis and diabetes.^1, 2^ FRDA is caused by a homozygous GAA repeat expansion mutation within intron 1 of the *FXN* gene.^3^ Unaffected individuals have 5–30 GAA repeats, whereas affected individuals have 70 to more than 1000 GAA triplets. The GAA expansion mutation reduces the expression of frataxin, a ubiquitous mitochondrial protein that is involved in iron-sulphur cluster (ISC) and haem biosynthesis.^4^ Evidence suggests that frataxin deficiency inhibits mitochondrial respiration and promotes production of reactive oxygen species (ROS), causing mitochondrial dysfunction, oxidative stress and subsequent mitochondrial iron accumulation.^4, 5^ These effects result in neuronal atrophy, where the primary sites of pathology are the dorsal root ganglia,^6^ and the dentate nucleus of the cerebellum.^7^ Previous studies of mitochondrial pathophysiology have been performed on post-mortem tissues or fibroblasts. However, mitochondria can be affected by the procedures of tissue extraction and conservation making these studies unreliable, whereas fibroblasts are not affected in FRDA and so pathological changes in these cells may not reflect underlying disease processes. The *Fxn* knockout mouse model has been shown to be embryonic lethal, and this has been followed by the development of conditional knockout mouse models specific for the central nervous system or the heart.^8^ Although useful for understanding some features of frataxin, these models could not be studied for one of the main features of the FRDA condition, which is the slow progression.^8^ We used a humanised mouse model, the YG8R transgenic mouse model, which contains a human *FXN* YAC with 190+90 GAA repeats on a mouse *Fxn* null background, that recapitulates the progressive disease phenotype shown in humans.^9, 10, 11, 12^ A similar approach has generated a control transgenic mouse that contains the same human *FXN* YAC, but with only nine GAA repeats, called Y47R mice.^13^ These mouse models have been validated and extensively used in studies on FRDA.^9, 14, 15^ Although mitochondrial dysfunction is believed to be one of the main causes of FRDA pathology, the effect of frataxin deficiency on mitochondrial function is not yet clear. The present study sought to investigate the changes in mitochondrial physiology in FRDA-like cerebellar granule neurons and glia, by using hemizygous YG8R mice (with a defective *FXN* transgene) and hemizygous Y47R mice (with a normal *FXN* transgene). The cerebellum is one of the most affected tissues in FRDA pathology^7, 16, 17, 18^ and cerebellar granule neurons have previously been shown to be lost in an inducible *Fxn* knockout FRDA mouse model.^19^ Although, patients show clear sings of cerebellar ataxia, it is not clear where the pathophysiology lies amongst the cerebellar neurons. In this work we aimed to investigate whether cerebellar granule neurons and glial cells, which are largely unexplored in FRDA, could be affected by the presence of the GAA repeat expansion and to investigate how frataxin deficiency could affect neuronal cell viability.

## Results

### YG8R cerebellar granule and glial cells show reduced frataxin levels

**Figure 1:**
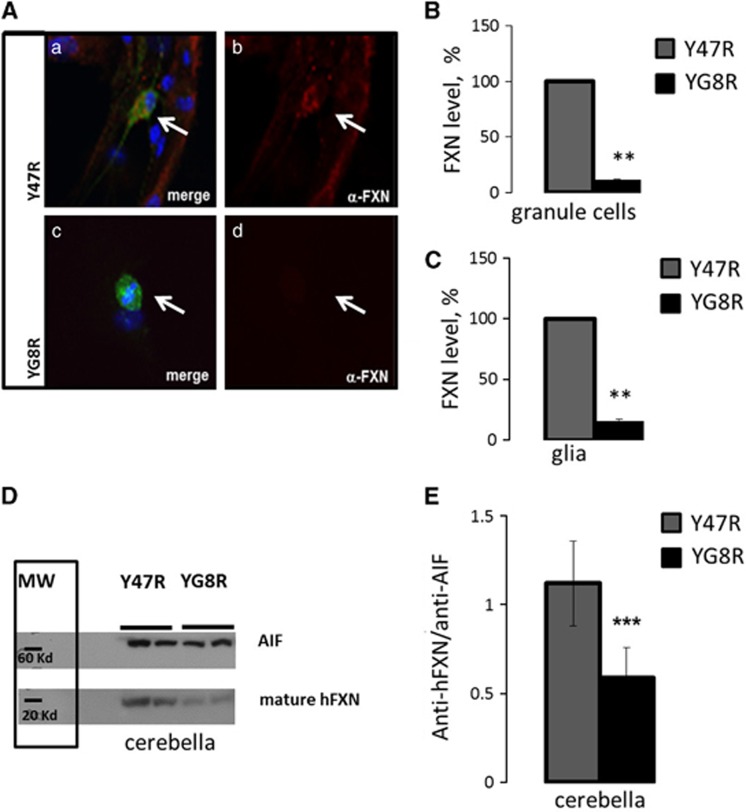
Decrease of FXN in granule cells and glia from YG8R mice. (**A**) The panel on the left shows immunofluorescence on co-cultures of granule cells and glia in the two genotypes: Y47R (control), YG8R (190 and 90 GAA repeats; FRDA model). In **a** and **c** are shown the merge of DAPI (blue), α-MAP-2 (green) and α-FXN (red), while **b** and **d** show α-hFXN (red). (**B**, **C**) The histograms represent the mean intensity of the α-hFXN antibody measured cell-by-cell. On top the histogram shows the mean of hFXN level in granule cells and on the bottom in glial cells. The fluorescence was averaged between cells and number of animals used (three independent experiments were conducted per case). In both cell types granule cells and glia, the level of FXN is significantly decreased in YG8R cells (**P<0.01; one–way ANOVA test with Bonferroni correction), compared with Y47R. (**D**) The figure shows the western blot on α-hFXN and α-AIF conducted on cerebella extracts of 8.5-months-old mice. (**E**) The histogram shows the quantification of the western blot (three independent experiments and *n*=3 mice; ***P=0.0004; *t*-test)

Frataxin levels were measured in co-cultures of cerebellar granule neurons and glial cells from Y47R and YG8R mice. Using immunofluorescence, we labelled human frataxin and measured the fluorescence intensity cell-by-cell, differentiating granule cells from glia with a neuronal marker (anti-MAP-2) (Figure 1A). We found that there is a significant decrease of frataxin in both cerebellar granule neurons and glial cells in the YG8R genotype, when compared to the Y47R (Figure 1B; granule cells YG8R 11.1%±1.3, *n*=97 cells; Figure 1C; glia YG8R 15.9±1.2, *n*=95 cells; **P<0.01; *n*=3 independent experiments).

The YG8R mouse model shows a molecular phenotype not earlier than 4 months of age and this is progressive as mice become older.^14^ Our experiments on primary cultures of neurons and glia show that the level of frataxin is representative of an adult phenotype of the mouse model. Indeed, when we measured the level of frataxin in the cerebellum of 8.5-month-old mice, we found that the level of frataxin is significantly decreased compared with the control (Y47R). The western blot image in Figure 1D shows a visible decrease in *hFXN* in YG8R cerebella compared with Y47R mice of the same age. The quantification of three independent experiments is represented in the histogram (Figure 1E; Y47R 1.12±0.24, YG8R 0.59±0.17; three independent experiments in duplicates of *n*=3 mice; ***P=0.0004), normalising the hFXN signal with a mitochondrial marker, the apoptotis-inducing factor protein (AIF).

Since it is known that frataxin is involved in the biosynthesis of haem and acts as a chaperon for ISCs,^20, 21^ its activity is crucial for those proteins that require ISCs to perform their functional activity,^22^ such as Complexes I and III of the mitochondrial electron transport chain (ETC) and aconitase.^14, 22^ As Complexes I and III are fundamental for the maintenance of the mitochondrial membrane potential (▵Ψ_m_), we investigated if mitochondrial respiration could be dependent on frataxin activity and therefore be affected by the decrease of this protein.

### YG8R cerebellar granule cells exhibit ▵Ψ_m_ abnormalities

**Figure 2:**
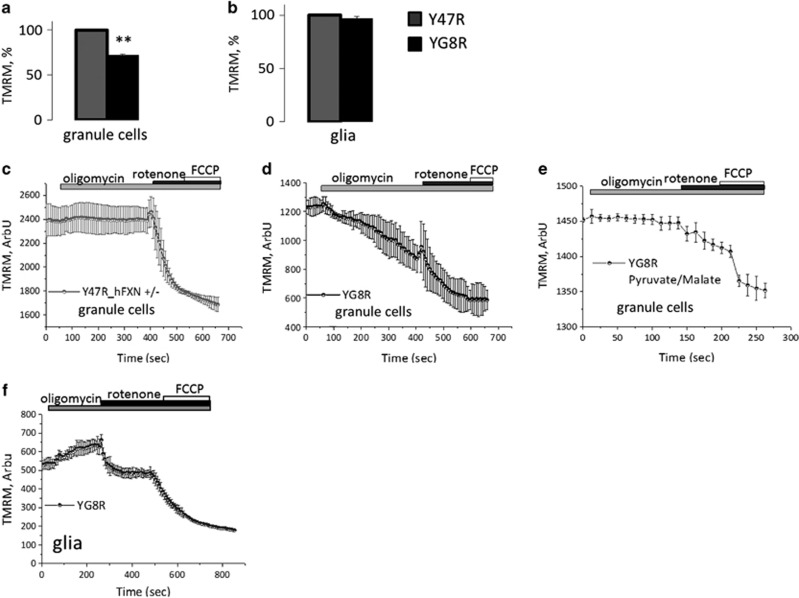
Study of Δψ_m_ in YG8R granule cells and glia. (**a** and **b**) The histograms represent the mean of the peak intensity value of TMRM (25 nM), taken cell-by-cell, and averaged between cells and number of animals used (no. of cells=150 per case). Although cerebellar granule neurons show a difference between Y47R (control) and YG8R (**P<0.005; one–way ANOVA test with Bonferroni correction), on the left side of the histogram (**a**), astrocytes do not seem to be affected by the presence of the mutated gene in YG8R (**b**). This is because of the fact that granule and glial cells have a different energy metabolism. (**c** and **d**) The graph shows the differential response to oligomycin (an inhibitor of Complex V of the ETC) from granule Y47R and granule YG8R; where YG8R cells show a decrease of potential during oligomycin, meaning that these cells cannot maintain their Δψ_m_ (no. of cells=100 per case). (**e**) The curve shows the response to oligomycin in granule YG8R cells treated with Complex I substrates (5 mM pyruvate and 5 mM malate for 12 h). (**f**) The curve shows the mean of responses from glial cells to oligomycin, from YG8R (**f**). In glial cells, YG8R show a normal response on regards to Δψ_m_ maintenance (no. of cells=140 per case)

▵Ψ_m_ is a unique indicator of mitochondrial health. By using tetramethylrhodamine (TMRM) fluorescence, we investigated whether cells expressing reduced frataxin could reveal differences in ▵Ψ_m_. The basal level of ▵Ψ_m_ in YG8R cerebellar granule neurons was significantly lower than Y47R cells (Figure 2a YG8R granule cells 72.01%±1.3, *n*=100 cells; **P<0.005), suggesting a crucial role of frataxin in ▵Ψ_m_ maintenance. Although the level of frataxin in YG8R glia cells is also very low (15.9% of the control), the ▵Ψ_m_ in these cells was similar for the two genotypes (Figure 2b; glia YG8R 98.03%±1.403, *n*=106 cells). This suggests a specific role of frataxin on mitochondria in granule cells or the presence of compensatory mechanisms in glia cells which maintain ▵Ψ_m_ in conditions of low frataxin. The ▵Ψ_m_ is normally generated by the respiratory chain. However, when this is compromised, the hydrolysis of ATP by the F1F0-ATPase (Complex V) will occur, pumping protons out of the mitochondria and thus maintaining the ▵Ψ_m_.^23^ To understand and explore the basis of the decrease of ▵Ψ_m_ in granule cells in our FRDA model, we examined the changes in ▵Ψ_m_ in response to specific mitochondrial inhibitors. The application of oligomycin (2 *μ*g/ml), the F1F0-ATPase inhibitor, induced no changes or small hyperpolarisation in Y47R granule compared with YG8R (Figure 2c and d). Importantly, in YG8R granule cells, the addition of oligomycin induced a slow and sustained decrease in ▵Ψ_m_ (Figure 2d). Consecutive addition of Complex I inhibitor, rotenone (5 *μ*M) induced, as expected, a profound mitochondrial depolarisation in all the cells, with complete depolarisation in YG8R granule cells (Figure 2d). These results suggest that in YG8R granule cells the ▵Ψ_m_ is mainly maintained by respiration. Addition of substrates for Complex I (5 mM pyruvate and 5 mM malate) 12 h prior to recording, induced a slight increase of the ▵Ψ_m_ in granule cells (Figure 2e). These results show that by giving Complex I substrate the loss of ▵Ψ_m_ in response to oligomycin can be rescued in YG8R granule cells, suggesting that the level of mitochondrial substrates could be the limiting factor for the respiratory chain in YG8R granule cells. In glial cells from YG8R cultures, the basal level of ▵Ψ_m_ was similar to Y47R; however, we examined ▵Ψ_m_ maintenance using the same experimental protocol as for granule cells. Investigating the ▵Ψ_m_ in glial cells, we found that the YG8R cells were responding as control (Figure 2f) corroborating the results looking at the basal level of ▵Ψ_m_, thus confirms that glial cells do not seem to be affected by the decreased level of frataxin, at least not in a bioenergetic manner as they can switch to glycolysis to sustain respiration.^24^

### Levels of NADH and FAD in granule cells from YG8R mice

Considering the difference in the level ▵Ψ_m_ of neurons and glia, we used a method that measures activity of mitochondrial respiration in single cells and tissue slices. The activity of the respiration in single cell or tissue slices can be estimated by measuring the autofluorescence of the major substrate for Complex I—NADH and FAD— to estimate the Complex II activity.

**Figure 3:**
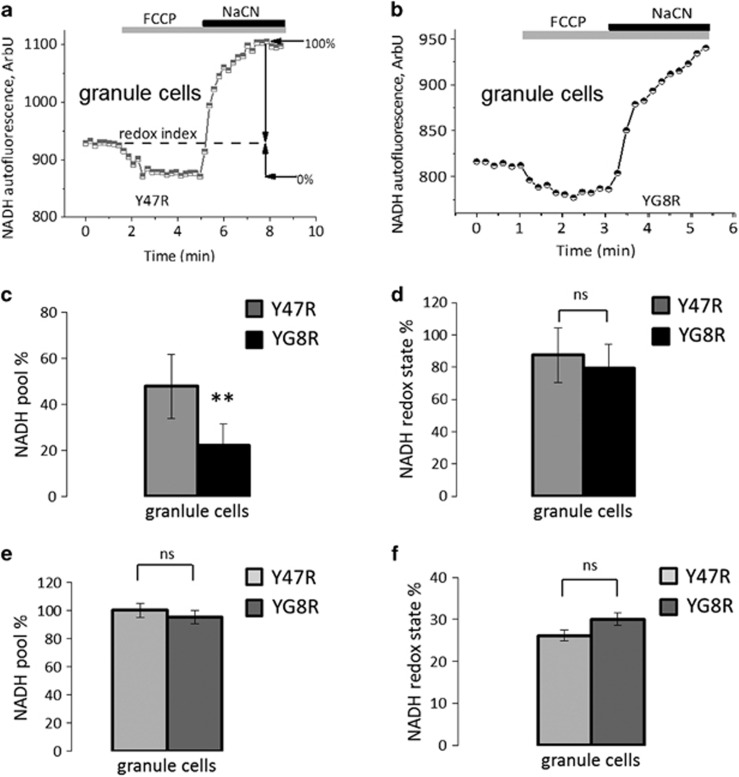
Investigation of NADH autofluorescence in cerebellar granule cells in culture and acute cerebellar slices. (**a** and **b**) The application of mitochondrial uncoupler 1 *μ*M FCCP maximises the rate of respiration and oxidises the mitochondrial NADH pool in cells, resulting in a decrease of detected fluorescence (minimum=0% for NADH; 3A; 3B). The subsequent application of the Complex IV inhibitor, 1 mM NaCN, suppresses respiration preventing NADH oxidation and allowing the NADH pool to be regenerated (maximum=100% for NADH; 3A; 3B) The first trace shows the response to 1* μ*M FCCP and 1 mM NaCN in a mean of granule cells Y47R, the second shows YG8R. (**c**). The histogram represents the NADH pool calculated from the traces above in both genotypes (**P=0.0057; Mann–Whitney test). (**d**) Shows the NADH redox level in granule cells. (**e** and **f**) Show respectively the NADH pool and the redox state in % in granule cells from acute slices, where no significant differences were found when comparing YG8R to Y47R

We found that the NADH pool in granule cells of YG8R is significantly lower than Y47R (Figure 3c; Y47R 47.69±0.045; YG8R 21.96±0.04; **P<0.005; and *n*=100 cells per genotype), which could be due to a lack of substrates for Complex I, that is in agreement with the ability of substrates to recover the ▵Ψ_m_ in these cells (Figure 2a). The NADH redox index (the balance between production of NADH in the tricarboxylic acid cycle (TCA) and oxidation in Complex I) was not significantly different between the two genotypes in granule cells from primary cultures (Figure 3d; Y47R 87.42±0.05; YG8R 79.35±0.053; *n*=100 cells per genotype). The NADH pool measured in acute slices, as opposed to single cells, did not show a significant decrease in YG8R in granule cells (Figure 3e; Y47R 100±.0.035; YG8R 95±0.04; *n*=3 mice per genotype). This suggests that in a more complicated and perhaps more physiological system, such as cerebellar slices, there is a form of compensatory effect from other components of the ETC. The NADH redox state was also unaltered in granule cells (Figure 3f; Y47R 26.1±0.048; YG8R 30±0.05; *n*=3 independent experiments) from acute slices isolated from aged (8.5 months) animals.

**Figure 4:**
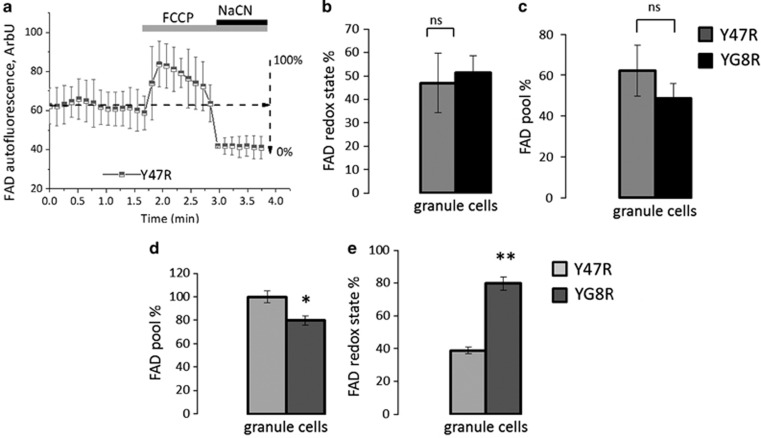
Investigation of FAD autofluorescence in cerebellar granule cells and acute cerebellar slices. (**a**) The application of mitochondrial uncoupler 1 *μ*M FCCP maximises the rate of respiration and oxidises the mitochondrial FADH_2_ pool in cells, resulting in an increase of detected fluorescence (maximum=100% for FAD; 4A). The subsequent application of the Complex IV inhibitor, 1 mM NaCN, suppresses respiration decreasing the level of FAD (minimum=−0% for FAD; 4A). The first trace shows the response to 1 *μ*M FCCP and 1 mM NaCN in a mean of primary cultures of cerebellar granule cells form Y47R looking at FAD autofluorescence. (**b** and **c**) The histograms represent the FAD pool calculated from the trace above in both genotypes in granule cells and the level of FAD redox state. No significant differences were detected between genotypes from primary cultures. (**d** and **e**) Show respectively the FAD pool and the redox state in granule cells from acute slices which were both significantly different, between Y47R and YG8R (*P<0.05 and **P<0.005)

We then looked at Complex II activity in primary cultures and acute slices measuring FAD autofluorescence cerebellar granule cells. Interestingly, the FAD pool and FAD redox state in granule cells from primary cultures (Figure 4a–c) were similar between the two genotypes (Y47R 47.02±0.03 of YG8R 51.35±0.026), indicating that Complex II was not compromised. By using the acute slices from aged mice we found that the FAD pool was significantly lower, reflecting an overactivation of the Complex II (Figure 4d; Y47R 100±0.052 of YG8R 79.8±0.046; *P<0.05; *n*=3 mice per genotype) and the FAD redox state was significantly higher in granule cells from FRDA-like mice (Figure 4e; Y47R 38.90±0.04 and YG8R 79.8±0.033; *n*=3 mice per genotype; **P<0.005).

### Mitochondrial respiration complex activities in YG8R cerebella

**Figure 5:**
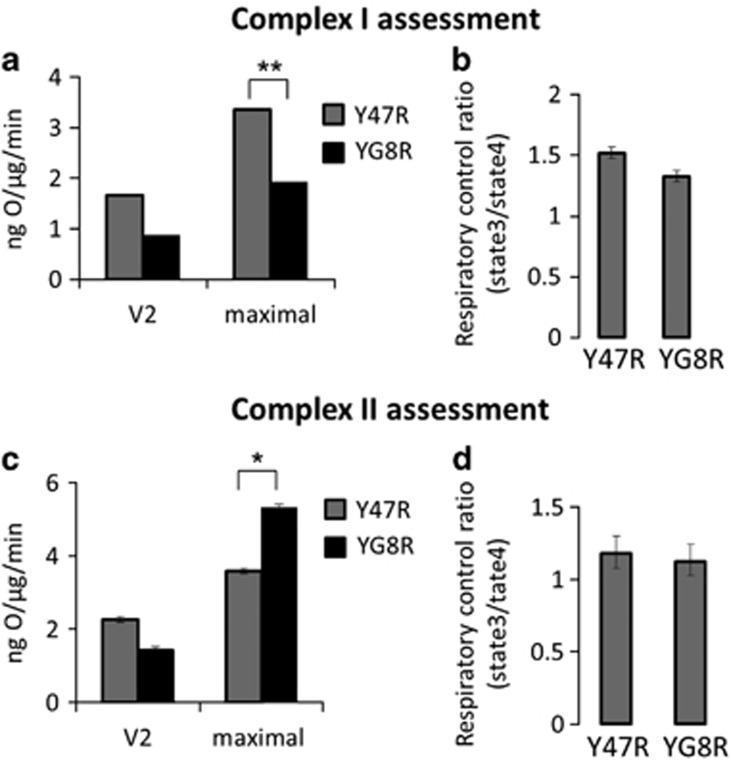
Oxygen consumption in mitochondria from cerebellum. (**a**) The histogram represent the rate of oxygen consumption originated from both Y47R and YG8R, showing the basal and the maximal oxygen consumption of isolated mitochondria from Y47R and YG8R cerebella, after administration of CI substrates (5 mM glutamate and 5 mM malate). The maximal level is significantly decreased in YG8R mitochondria (**P<0.005). (**b**) The histogram represents the respiratory control calculated by state 3 divided to state 4, no significant differences were witnessed. (**c**) The graph shows basal and the maximal oxygen consumption of isolated mitochondria from Y47R and YG8R cerebella, after administration of the CII substrate (5 mM succinate) and 10 *μ*M rotenone (CI inhibitor) to exclude CI activity. The maximal level shows a significant increase in YG8R compared with the control (*P<0.05). (**d**) The histogram represents the respiratory control calculated by state 3 divided to state 4, showing no significant differences between the two genotypes

To further investigate mitochondrial function in our models, we studied the effect of the YG8R genotype on oxygen consumption in isolated mitochondria from cerebella. The basal rate of respiration (V2) of YG8R mitochondria in the medium containing substrates for Complex I (5 mM glutamate/5 mM malate) was slower than control values (Figure 5a and b; compare 1.6±0.01 in Y47R to 0.86±0.012 in YG8R*, n*=3 mice). The maximal rate of respiration can be stimulated by uncoupler FCCP (0.5 *μ*M). We have found that the maximal rate of respiration in glutamate/malate medium was significantly less in YG8R (compare 1.9±0.017 to 3.4±0.14 in control, *n*=3; **P<0.005; Figure 5c). Importantly, the respiratory control ratio (the ratio between ADP dependent (V3) and ADP independent respiration) was similar for both genotypes in glutamate/malate (Y47R 1.5±0.037; YG8R 1.32±0.04; Figure 5b). Experiments conducted in the medium with substrate for Complex II (5 mM succinate plus inhibitor of Complex I 5*μ*M rotenone) produced results that were directly opposite, that is, an increase of the maximal rate in FRDA-like cerebella (Fig. 5c; 5.3±0.08 in YG8R compared with control 3.5±0.07; *n*=3; **P<0.005). The respiratory control ratio, in the presence of succinate and rotenone, was similar for both genotypes (Y47R 1.17±0.1; YG8R 1.12±0.12; Figure 5d) suggesting the absence of mitochondrial uncoupling in YG8R mitochondria. These results strongly suggest the inhibition of Complex I in YG8R mitochondria with compensatory activation of Complex II.

**Figure 6:**
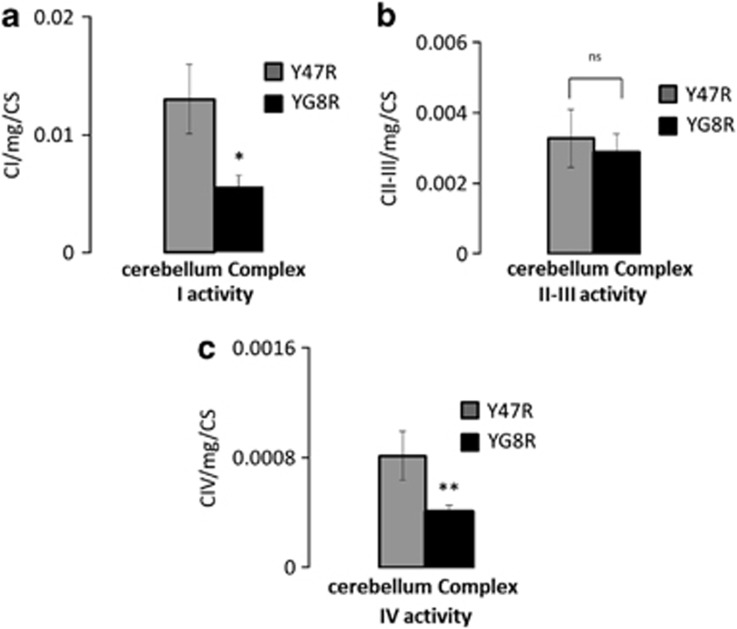
Mitochondrial complex activities in cerebellum of YG8R mice. (**a**) The histogram shows the activity of CI measured from cerebellar homogenates, which results decreased in YG8R mice (*P=0.02). (**b**) Shows the activity of both CII–III which is not significantly different between the two genotypes. (**c**) The histogram shows the activity in CIV in Y47R and YG8R cerebellar homogenates. YG8R shows a significant decrease of activity (**P=0.008)

Therefore, we further investigated the activity of the ETC Complexes (CI, CII–III and CIV) in cerebellar homogenates (Figure 6a–c). Data were normalised by total protein concentration and citrate synthase activity was used as a mitochondrial activity marker. In YG8R mice, CI activity was significantly lower than in Y47R mice (Figure 6a; Y47R 0.013±0.003; YG8R 0.005±0.0009; *n*=8 mice; *P=0.02). As suggested by our previous experiments the level of CII–III activities in YG8R mice was not significantly different compared with Y47R mice (Figure 6b; Y47R 0.003±0.0008; YG8R 0.002±0.0005; *n*=8 mice), confirming that CII is not impaired. Interestingly, CIV activity in YG8R mice was significantly lower than Y47R mice (Figure 6c; Y47R 0.0008±0.0002; YG8R 0.0004±5.1e−05; *n*=8 mice; **P=0.008). In the case of Complex IV inhibition molecular oxygen coming from Complex III cannot be converted into H_2_O and that could also increases ROS.

### Mild mitochondrial impairment causes generation of ROS

**Figure 7:**
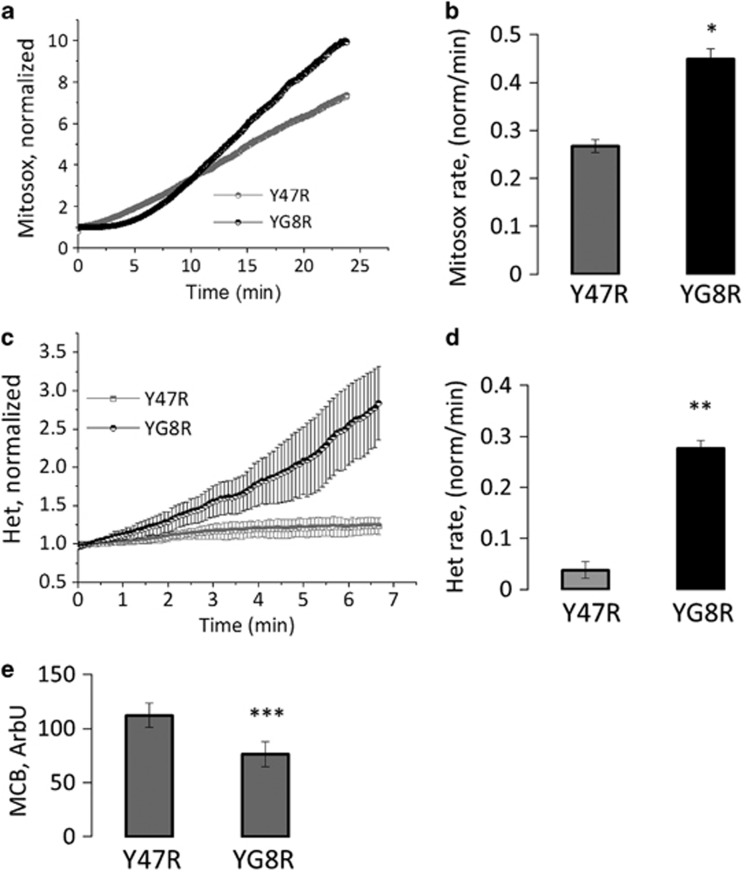
ROS increases and GSH decrease in cerebellar granule cells of FRDA-like cultures. (**a** and **b**) Mitochondrial ROS were measured with Mitosox in cerebellar granule cells. The curves on the left show the increase over time of Mitosox fluorescence (**a**), which was quantified cell-by-cell as a rate of mROS generation (**b**). YG8R showed a significant increase in rate of mROS generation (*P<0.05). (**c** and **d**) Similarly to the mROS also the cytosolic ROS were higher in the FRDA-like granule cells. Cytosolic were measured with dihydroethidium (Het). The level of ROS production is visible with the Het kinetic over time (**c**) and the rate showed a significant increase of ROS (**d**; **P<0.005). (**e**) By using monochlorobimane (MCB) we have measured the level of GSH in cerebellar granule neurons, which showed a significant difference between YG8R and control (***P=0.0001)

**Figure 8:**
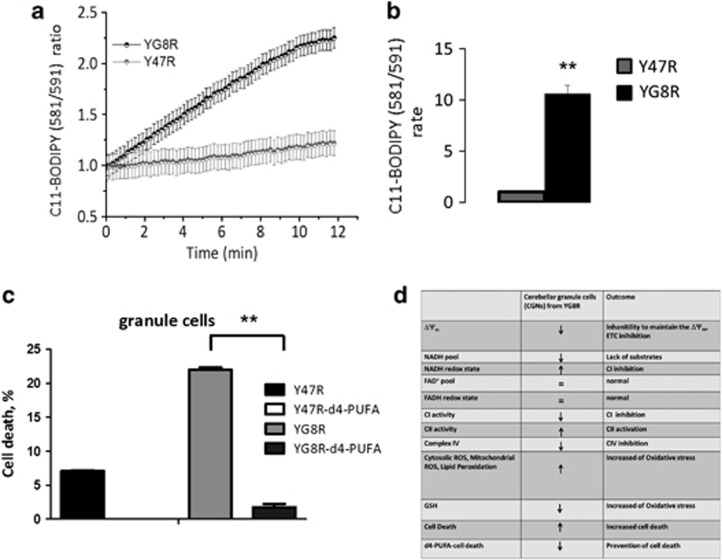
Lipid peroxidation is increased in YG8R and anti-lipid peroxidation protects YG8R from cell death. (**a**) The graphs show respectively the kinetic ratio from Y47R and YG8R, using C11-BODIPY and (**b**) shows the rate of lipid peroxidation in granule cells from Y47R and YG8R. The FRDA-like genotype results in a significant increase of lipid peroxidation compared to control (**P<0.005). (**c**) The histogram shows the percentage of cell death in YG8R cerebellar granule cells, with and without 24 h treatment with 100 *μ*M D_4_-PUFAs. This compound resulted to be protective showing a significant decrease in cell death (**P<0.005). (**d**) Summary of the results

Partial and complete inhibition of the Complex I can result in excessive ROS production, which may be a reason for cell death.^25, 26, 27^ Figure 7 shows an increase of mROS measured with mitosox in Y47R and YG8R. The rate of mROS is significantly higher in YG8R granule cells (Figure 7a and b; Y47R 0.48±0.01; YG8R 0.27±0.02; *P<0.05; *n*=3 independent experiments). We also found that cytosolic ROS was increased in YG8R granule cells (Figure 7c and d; Y47R 0.03±0.016; YG8R 0.27±0.08; **P<0.05; *n*=3 independent experiments). If ROS are not counteracted by scavengers and antioxidants, the excessive ROS generation can result in oxidative stress that leads to oxidation of lipids. To investigate the effect of decreased frataxin on the level of the major neuronal antioxidant glutathione, we used a specific probe, monochlorobimine. We found that excessive ROS generation from the mitochondria and the cytosol of YG8R cerebellar granule cells led to a significantly decreased level of reduced glutathione (GSH) compared with controls (Figure 7e; Y47R 112±20.9; YG8R 76±11.5; ***P<0.0005; *n*=3 independent experiments). To estimate the rate of lipid peroxidation in the Y47R and YG8R cells, we used the indicator C11-BODIPY (581/591). We found that the rate of lipid peroxidation in YG8R cells was 10.6-fold higher than Y47R cells (Figure 8a and b; YG8R 10.6±0.02; **P<0.005; *n*=3 independent experiments). The dramatic increase of lipid peroxidation suggests that this may be a crucial effect of frataxin silencing. To confirm that the lipid peroxidation has a central role in frataxin-deficient cells, we have challenged cells with a novel compound that counteracts lipid peroxidation and looked at cell death.

### Cell death in granule cells from YG8R mice

Since lipid peroxidation was most severely increased (10-fold) in YG8R granule cells, we assessed cell death with and without the presence of a compound called d4-PUFA, which has been shown to counteract the oxidation of lipids *in vitro*.^28^ By measuring cell death in granule cell cultures with propidium iodide (PI), we assessed the level of death in untreated cells compared with cells treated with 100 *μ*M d4-PUFA (24 h incubation). Figure 8c shows that the compound remarkably prevented YG8R cell death (YG8R 21.9±0.34; d4-PUFA-YG8R 1.75±0.52; and *n*=3 independent experiments). Interestingly, Y47R cells do not show the physiological basal level of cell death, demonstrating that d4-PUFA is also beneficial in control cultures. This result suggests that the mild bioenergetic impairments lead to an increase of ROS that immediately generate peroxidation of lipids in the vicinity of PUFA-rich mitochondrial membrane and throughout the cell.^29, 30^

## Discussion

Although the role of frataxin is largely known, being fundamental for the iron biogenesis in the cell, the relation between frataxin and mitochondrial bioenergetics is not completely clear. Here, we demonstrate the close participation of frataxin in maintaining healthy mitochondrial physiology of cerebellar granule neurons. For the first time, using a validated FRDA model, we have investigated mitochondrial physiology with functional microscopy techniques. We found that, the frataxin-deficient YG8R mouse model showed a limitation of the maintenance of ▵Ψ_m_ in cerebellar granule neurons, with a specific deficiency in Complex I. Complex I substrates, such as pyruvate and malate, incubated for 12 h, prevent the ▵Ψ_m_ maintenance defects observed in YG8R cerebellar granule cells. The NADH pool in primary cultures of granule cells is significantly decreased in YG8R cultures, even if the NAD^+^/NADH redox state is not significantly different to the control. The dramatic decrease of ▵Ψ_m_ seems to be caused by a lack of NADH availability for Complex I to work. This could be due to a normal consumption of NADH by the TCA cycle, which cannot be regenerated by new NAD^+^ reduction due to other proteins usage. One of the possible candidates could be PARP-1, which uses NAD^+^ during severe oxidative stress^31^ and could affect the action of sirtuin proteins.^32^ The crucial function of sirtuins has been revealed due to their inactivation in FRDA models,^33^ causing massive hyperacetylation and genomic instability.^34^ This inactivation is linked to NAD^+^ deprivation (as a limiting factor of sirtuin activities) and excess of nicotinamide, which are both effects of PARP-1 overactivity.^32^ This should be the object to future investigations. By looking at acute cerebellar slices, the level of NADH does not seem to be particularly affected in cerebellar granule neurons. However, the redox state of FAD is significantly increased, demonstrating that Complex II is overworking. These results were also confirmed with two other different techniques: firstly by measuring oxygen consumption in isolated mitochondria, and secondly by measuring Complex activities. The first technique showed that while the maximal respiration for Complex I decreases, Complex II is increased. This explains why respiratory control does not seem to change between the two genotypes. It also indicates that Complex I has a mild impairment and that Complex II is slightly overworking to compensate the respiration. Indeed the respiratory control is not significantly defective. By investigating the Complex activities we confirmed that Complex I is less active and that Complexes II–III are not affected. Complex IV also demonstrated compromised activity, indicating that the increase of ROS may also be due to an impairment of this Complex. The inhibition of Complex IV was found in FRDA patient's lymphoblasts and that could perhaps not only be induced by a lower activity of the Complex III but also from a deficiency present in Complex IV due to lower frataxin and the haem chain impairments.^35^

Inhibition of Complex I with activation of Complex II can stimulate the reverse flux of electrons and the production of ROS on both sides of the mitochondrial membrane.^36^ Here we have demonstrated that the levels of mitochondrial and cytosolic ROS are increased in YG8R cerebellar granule neurons and result by the massive increase of lipid peroxidation. Excessive ROS production results in oxidative stress and reduces the level of GSH. We also proved that GSH is decreased in granule cells, indicating that there is probably a downregulation of the GSH antioxidant pathway, similar to previous observations in other yeast and human lymphoblast cell models.^37, 38^ During oxidative stress, the most damaging by-products are peroxidized lipids.^28^ Moreover, as consequences of mitochondrial dysfunction and oxidative stress, lipid peroxidation and its lipid-derived neurotoxins are considered to be one of the major causes of neurodegeneration, since the CNS is especially enriched in polyunsaturated fatty acids compared to other systems^29, 39^ (Figure 8d).

We have recently described that this cascade of events happens also in fibroblasts from YG8R and KIKO,^40^ another validated FRDA models, and by using Nrf2-inducers we rescued both the mitochondrial phenotype and prevented the increase of lipid peroxidation.^41^ The activation of Nrf-2 pathway, indeed, not only triggers the increase of endogenous GSH^42^ but also regenerates the substrates for Complexes I and II of the mitochondrial respiratory chain.^43^ In FRDA models, such as drosophila and mouse fibroblasts, it has been found that the by-products of lipid peroxidation are massively increased.^41, 44, 45^ By looking at lipid peroxidation, specifically in granule cells, we found a dramatic increase in YG8R compared to Y47R. Since lipid peroxidation is one of the most toxic effects of oxidative stress in the CNS, we assessed whether this could be a source of premature cell death in YG8R granule cells. Cultures of granule cells were treated with d4-PUFA, a compound known to prevent lipid peroxidation.^28, 46, 46^ The reduction of lipid peroxidation protected these granule cells, strongly implicating oxidative stress as a major reason for degeneration in the mild form of frataxin depletion.

In conclusion, we have studied cerebellar neuronal cells in the YG8R FRDA mouse model, which presents with a slowly progressive phenotype, similar to late-onset FRDA patients. We assessed the type of mitochondrial dysfunction that was present in the cerebellum, concluding that Complex I activity is impaired, but Complex II compensates by overworking. Therefore, if we consider the ETC, we can define the mitochondrial dysfunction as a mildly defective bioenergetic phenotype. However, this mild dysfunction drives the formation of free radicals that cannot be attenuated by the endogenous antioxidant systems, which are downregulated. Thus, the level of lipid peroxidation increases dramatically, damaging the cells and causing premature cell death. We have presented for the first time a full description of the mitochondrial pathophysiological behaviour in the YG8R mouse model and we have proven that the lipid peroxidation is the major cause of cell toxicity in this model. Furthermore, we have shown that by counteracting lipid peroxidation with d4-PUFA, in cerebellar granule cells, we can prevent neuronal death. This was also confirmed recently on fibroblasts of FRDA mouse models.^41^ Therefore, lipid peroxidation could be a potential target for future therapeutic approaches in FRDA.

## Materials and Methods

### Cerebellar granule neuronal cultures

Primary cultures of cerebellar granule neurons were obtained from cerebella of 6-day-old YG8R and Y47R mice.^13, 14^ Cerebella were triturated and then incubated with 0.25% Trypsin EDTA solution (Sigma-Aldrich, Gillingham, UK) for 15 min at 37 °C. The homogenates were centrifuged at 1000 r.p.m. for 4 min. Then, the tissues were washed with HBSS w/o Ca^2+^ and Mg^2+^ and centrifuged twice before adding DMEM-glutamax at 10% fetal bovine serum, and penicillin and streptomycin. Cells were seeded on glass coverslips and after 5 h Neurobasal A, B27, penicillin and streptomycin and l-glutamine. The coverslips were pre-coated with Poly-d-lysine (1 mg/ml). Cells were kept in Neurobasal A, glutamine, antibiotics and 25 mM KCL. Ara-C was added within 24 h from the plating to prevent the over growth of non-neuronal cells. Neurons were used after 9/10 days for all the experiments.^47^

### Cerebellar slices

Cerebella were freshly isolated and placed immediately in ice-cold HEPES-buffered salt solution (HBSS) composed (mM): 156 NaCl, 3 KCl, 2MgSO4, 1.25 KH_2_PO_4_, 2 CaCl_2_, 10 glucose and 10 HEPES, pH 7.35 (HBSS 1 ×) and sliced at 1 °C using a vibratome (Leica VT1200S, Milton Keynes, UK). Transverse acute cerebellar slices (~100 *μ*m) were prepared from 8.5 months old Y47R and YG8R mice. The tissue slices were cut and maintained in HBSS 1 × at 25 °C room temperature (RT) for ~1 h before imaging.

### Immunofluorescence

Primary co-cultures of cerebellar granule neurons and glia, plated on glass coverslips, were fixed with ice-cold 4% (v/v) paraformaldehyde in phosphate-buffered saline (0.2 M Na_2_HPO_4_ adjusted with 0.2 M NaH_2_PO_4_ to pH 7.4) for 30 min and subsequently permeabilized for 15 min using 0.5% (v/v) Triton X-100 in phosphate-buffered saline (PBS 1 ×). After a blocking step, with 3% BSA and 10% goat serum dissolved in PBS 1 × and 0.1% Triton X-100, for 1 h at RT, the cells were incubated with primary antibodies. Human frataxin was detected by using a mouse monoclonal antibody (Abcam, Cambridge, UK) diluted 1:100. Neurons were identified using chicken anti-MAP-2 (1:1000, Abcam). All the antibodies were diluted in blocking solution and incubated overnight at 4 °C. Cells were washed with PBS 1 ×, and followed by 1 h incubation with the secondary antibody at RT. Anti-mouse Alexafluor 565 (1:500) and anti-chicken Alexafluor 488 (1:500) in blocking solution. 300 nM of DAPI was incubated for 5 min and washed with PBS 1 ×. Coverslips were mounted with Dako (Ely, Cambridgeshire, UK) mounting medium.

### Western blotting

Isolated mitochondria from cerebella were resuspended in ice-cold RIPA 1 × buffer (Sigma-Aldrich), with inhibitors of protease (Roche, West Sussex, UK). After solubilisation, the samples were centrifuged at maximum speed at 4 °C. BCA assay (Thermoscientific, Loughborough, UK) was applied to all the samples. Loading buffer was added in a 1:1 ratio with the samples and boiled samples at 100 °C for 10 min, and loaded onto a 10% acrylamide gel. The electrophoresis was blotted to PVDF membrane. The blotting was done with mouse α-hFXN (1:1000; Abcam) and rabbit α-AIF (1:1000; Abcam). Secondary HRP-conjugated antibodies were used at a 1:5000 dilution. Analysis was done with Image J software (Bethesda, MD, USA).

### Mitochondrial membrane potential assay

Mitochondrial membrane potential (▵Ψ_m_) was measured with tetramethyl rhodamine methyl ester (TMRM, 25 nM, Invitrogen) in 'redistribution mode':^48^ the dye was allowed to equilibrate and was present continuously in the recording solution. TMRM distributes between cellular compartments in response to different potentials and, at concentrations <50 nM, in healthy cells the fluorescent signal shows a mitochondrial localisation, where is retained until mitotoxins induced depolarisation. The basal level of ▵Ψ_m_ was measured by exciting TMRM at 560 nm and collecting the images with a 590 nm long-pass filter. With Z-stacks configuration the fluorescence peaks from the mitochondrial network were collected and analysed. The maintenance of ▵Ψ_m_ was measured after using 2 *μ*g/ml oligomycin, 1 *μ*M rotenone and 1 *μ*M Fccp.

### NADH autofluorescence

The autofluorescence of NADH and NADPH (which can be referred to NAD(P)H) in cerebellar granule neurons cultures was imaged on a Zeiss 510 META UV–vis confocal microscope (Cambridge, UK). The blue autofluorescence emitted by the pyridine nucleotides NADH and NADPH in their reduced form was excited with a UV laser (Coherent; at 351 nm; minimal laser power) and emission was collected using a 435–485 nm band pass filter. To measure the dynamic range of the signal in relation to the full-mitochondrial NADH pool and to normalise the data, the maximum oxidation and maximum reduction, cells were exposed to carbonyl cyanide 4-(trifluoromethoxy) phenylhydrazone (FCCP, 1 *μ*M—to stimulate respiration and achieve maximum NADH oxidation) and NaCN (1 mM—to inhibit respiration and achieve maximum NADH reduction). The application of mitochondrial uncoupler 1 *μ*M FCCP maximises the rate of respiration and oxidises the mitochondrial NADH pool in cells, resulting in a decrease of detected fluorescence (minimum=0% for NADH; Figure 3a and b). The subsequent application of the Complex IV inhibitor, 1 mM NaCN, suppresses respiration preventing NADH oxidation and allowing the NADH pool to be regenerated (maximum=100% for NADH; Figure 3a and b).

The final formula used to normalise the NADH autofluorescence measurement was: ▵*F*−*F*_fccp_=▵*F*_NaCN_−*F*_fccp_.^31^ Quantitative analysis of the images obtained was done using the Zeiss LSM 510 software.

### FAD autofluorescence

The autofluorescence of FAD in cerebellar granule neurons cultures was imaged on a Zeiss 710 confocal microscope. The green autofluorescence emitted by the flavoproteins FAD in their oxidised form was excited with an argon laser (Coherent; at 488 nm) and emission was collected after 510nm. To measure the dynamic range of the signal in relation to the full-mitochondrial FAD pool and to normalise the data, cells were exposed to FCCP, 1 *μ*M—to stimulate respiration and achieve maximum FAD oxidation, accompanied to an increased fluorescence) and NaCN (1 mM—to inhibit respiration and achieve maximum FAD reduction, accompanied to an decreased fluorescence).

The application of mitochondrial uncoupler 1 *μ*M FCCP maximises the rate of respiration and oxidises the mitochondrial FADH_2_ pool in cells, resulting in an increase of detected fluorescence (maximum=100% for FAD; Figure 4a). The subsequent application of the Complex IV inhibitor, 1 mM NaCN, suppresses respiration preventing FADH_2_ oxidation decreasing the fluorescence signal (minimum=0% for FAD; Figure 4a).

The formula used to normalise the FAD autofluorescence measurement was: ▵*F*−*F*_NaCN_=▵*F*_fccp_−*F*_NaCN_.^31, 43^ Quantitative analysis of the images obtained was done using the Zeiss Zen software.

### Oxygen consumption in isolated mitochondria

To measure respiration rate in isolated mitochondria from cerebella were extracted from fresh tissue with isolation buffer (250 mM Sucrose, 5 mM Tris/HCl, 2 mM EGTA pH=7.4/7.2 and 1% BSA). The cerebella were homogenised in a Teflon-glass homogeniser and resuspended in the mitochondrial isolation buffer. The homogenates were centrifuged at 6000 rpm at 4 °C for 11 min and then the supernatant was transferred into a new tube and ultracentrifugated at 20 000 r.p.m. for 15 min at 4 °C. The pellet was then resuspended in 200 *μ*l of isolation buffer and kept on ice until the beginning of the experiments. The recording medium consisted of: 135 mM KCl, 10 mM NaCl, 20 mM Hepes, 0.5 mM KH_2_PO_4_, 1 mM MgCl_2_, 5 mM EGTA, 1,86 mM CaCl_2_. A total of 5 mM pyruvate and 5 mM malate were added at the beginning of the recording when complex I activity was assessed. 5 mM Succinate and 10 *μ*M Rotenone were added at the beginning of the experiment when complex II activity was assessed. Experiments were conducted in a Clark-type oxygen electrode thermostatically maintained at 25 °C. The oxygen electrode was calibrated with air-saturated water, assuming 406 nmol Oatoms/ml at 25 °C (Oxytherm system, Hansatech Instruments, Norfolk, UK). The rate of oxygen consumption was measured using 50 nM ADP (state 3=V3), 2 *μ*g/ml Oligomycin (state 4=V4) and 0.5 *μ*M Fccp, which was added at the end of every experiment to establish maximal uncoupled respiratory rate.

### Complex activities

Cerebella were freshly isolated and immediately placed in mitochondria isolation buffer: constitute of 320 mM sucrose, 1 mM EDTA and 10 mM Trizma-base. Samples were homogenised using a hand-held ground glass homogeniser (Jencons Scientific Ltd, Bedfordshire, UK) using 1 g of tissue per 9 ml of isolation buffer. Protein and mitochondrial enzymes were assayed spectrophotometrically using a Uvikon XL spectrophotometer (Uvikon, Potton, UK). Mitochondrial membranes were disrupted using three freeze-thaw cycles in liquid nitrogen and a 30 °C water bath. Protein concentration was determined using the method of Lowry using a Folin-Ciocalteu reagent (Bio-Rad Laboratories Ltd, Hertfordshire, UK) containing a phosphomolybdic-tungstic mixed acid, to form a blue chromogen detected with λ_max_ of 750 nm. Complex I activity was determined by monitoring the disappearance of NADH as it is oxidised to NAD^+^ with *λ*_max_ of 340 nm and endogenous non-specific complex I activity subtracted after blocking with rotenone. Complexes II–III activities were determined by monitoring the succinate-dependent antimycin-A-sensitive reduction of cytochrome c with *λ*_max_ of 550nm. Complex IV activity was determined by measuring the oxidation of reduced cytochrome c by cytochrome oxidase with *λ*_max_ of 550 nm. Citrate synthase activity was determined using the^49^ assay. Enzyme activities are expressed as a ratio to CS (mitochondrial marker enzyme) to compensate for mitochondrial enrichment in each sample^50^ and total protein values.

### Imaging ROS generation and lipid peroxidation

MitoSOX (10 *μ*M) was loaded for 10 min at RT, and then imaged with 488 nm laser and long-pass 530-nm emission filter, to assess mitochondrial ROS (mROS). To measure cytosolic ROS generation of rates of ROS generation in the cytosol with dihydroethidium (Het) (5 *μ*M) the dye was present in all solutions throughout the experiments. No preincubation was used to preserve the compound from early oxidation.^51^ Het was excited at 530 nm and emissions were collected with a 560 nm long-pass filter, using a Zeiss 710 CLSM confocal microscope. Lipid peroxidation was estimated by using C11-BODIPY (5 mM; Molecular Probes, Loughborough, UK). Cells were incubated with 10 *μ*M C11-BODIPY (581/591) for 10 minutes and RT. C11-BODIPY was excited using the 488 and 563 nm laser line, and fluorescence measured from 505 to 550 nm and 570 and 630 nm. Fluorescence was measured using a Zeiss 710 CLSM confocal microscope.

### Glutathione measurements

To measure glutathione concentration (GSH), cells were incubated with 50 *μ*M monochlorobimane (MCB) in HBSS at room temperature for 40 min, or until a steady state had been reached before images were acquired for quantitation.^52, 53^ The cells were then washed with HBSS, and images of the fluorescence of the MCB-GSH adduct were acquired using a cooled CCD imaging system as described using excitation at 380 nm and emission at 400 nm.

### Cell death

Cells were treated 24 h with 100 *μ*M d_4_-PUFA. Prior imaging, cells were incubated with propidium iodide (PI; 10 *μ*M) and 300 nM DAPI for 15 min, washed 3x with PBS1x and analysed using a cooled CCD camera. DAPI stains all nuclei while PI stains only cells with a disrupted plasma membrane. Dead cerebellar granule neurons (PI positive), were counted as a fraction of the total. In each experiment, >200 neurons were examined in random fields from three independent cultures for each condition.

### Statistical analysis

Statistical analysis was performed with the aid of Origin 9 (Microcal Software Inc.) software. Results are expressed as means±S.E.M. The ANOVA test was employed when appropriate and the point of minimum acceptable statistical significance was taken to be 0.05, and was Bonferroni corrected where required. Mann–Whitney *U*-test was used when independent experiments were compared.

## Abbreviations

AIF: Apoptosis inducing factor
CCD: Charged coupled device
CI: Complex I
CII: Complex II
CIII: Complex III
CIV: Complex IV
CV: Complex V
ETC: Electron transport chain
FAD: *flavin adenine dinucleotide oxidised*
FADH2: *flavin adenine dinucleotide reduced*
FCCP: carbonyl cyanide 4-(trifluoromethoxy) phenylhydrazone
FRDA: Friedreich's Ataxia
GSH: Glutathione
HBSS: Hanks' Balanced Salt solution
ISC: Iron sulphur clusters
MCB: Monochlorobimane
NAD: Nicotinamide adenine dinucleotide oxidised
NADH: Nicotinamide adenine dinucleotide reduced
GAA: Glutamine
*FXN*: Frataxin gene
▵Ψm: Mitochondrial membrane potential
MCB: Monochlorobimane
NADH: Nicotinamide adenine dinucleotide reduced
NAD: Nicotinamide adenine dinucleotide oxidised
Nrf2: Nuclear factor (erythroid-derived 2)-like 2
PARP1: Poly (ADP-Ribose) Polymerase 1
PI: Propidium iodide
PUFA: Polyunsaturated fatty acids
ROS: Reactive Oxygen species
SIRT1: NAD-dependent deacetylase sirtuin-1
TCA: tricarboxylic acid cycle
YAC: *Yeast artificial chromosomes*
Y47R: Control mouse line
YG8R: FRDA-like mouse line
